# MRI characteristics of intracranial masses in the paediatric population of KwaZulu-Natal: A neuroimaging-based study

**DOI:** 10.4102/sajr.v25i1.2042

**Published:** 2021-05-28

**Authors:** Nompumelelo P. Gumede, Sithembiso M. Langa, Basil Enicker

**Affiliations:** 1 Department of Radiology, College of Health Sciences, University of KwaZulu-Natal, Durban, South Africa; 2 Department of Radiology, Jackpersad and Partners Inc., Durban, South Africa; 3 Department of Neurosurgery, College of Health Sciences, University of KwaZulu-Natal, Durban, South Africa

**Keywords:** intracranial masses, brain tumours, brain abscess, tuberculosis, magnetic resonance imaging

## Abstract

**Background:**

MRI is the imaging modality of choice for the assessment of intracranial masses in children. Imaging is vital in planning further management.

**Objectives:**

The purpose of this study was to describe the common intracranial masses and their imaging characteristics in the paediatric population referred to Inkosi Albert Luthuli Central Hospital for MRI of the brain.

**Method:**

We retrospectively reviewed the medical records of paediatric patients (aged from birth to 18 years) who underwent MRI investigations for intracranial masses between January 2010 and December 2016.

**Results:**

A total of 931 MRI brain scans were performed. One hundred and seven scans met the inclusion criteria, of which 92 were primary brain tumours and 15 were inflammatory masses. The majority were females (56%). The mean age was 12 ± 4.52 (range of 3–18 years). The most common presenting symptom was seizures (70/107, 65.4%). We categorised the masses according to supra- and infratentorial compartments. The most common site for masses was the supratentorial compartment (*n* = 56, 52%). The most common masses in the supratentorial compartment were craniopharyngiomas (14/45, 31.1%), whilst in the infratentorial compartment, the most common masses were medulloblastomas (24/47, 51.1%).

**Conclusion:**

In our series, the supratentorial compartment was the commonest site for intracranial masses. The most common tumour in the infratentorial compartment was medulloblastoma. This information is vital in formulating differential diagnoses of intracranial masses.

## Introduction

In low- and middle-income countries (LMICs) such as South Africa, brain tumours are the second most common tumours in the paediatric population, after leukaemia.^1,2,3^ They are responsible for the most common cancer-related deaths in paediatric patients.^2,3^ In high-income countries, brain tumour incidence ranges from 1.15 to 5.14 cases per 100 000 children, with the highest rates reported in the United States.^4^ The recent South African tumour registry dated 1997–2007 reported the annual incidence of tumours in children aged 0–14 years to be between 33.4 and 47.2 per million from 2003 to 2007.^1^ Brain tumours represented 13.45% of all diagnosed childhood tumours.^1^ Overall, the most common paediatric tumour in the supratentorial compartment is astrocytoma (68%), followed by craniopharyngioma (50%). Medulloblastoma (35% – 40%) and pilocytic astrocytoma (30%) are the common tumours in the infratentorial compartment.^5,6^

Intracranial infections are a major burden in LMICs because of poverty, overcrowding, inadequate access to clean water and proper sanitation systems, and insufficient access to healthcare. In a recent meta-analysis study by Robertson et al., sub-Saharan Africa had the highest rates of bacterial meningitis, neurocysticercosis and tuberculosis-related disease, with an incidence of 65 to 650 per 100 000. This is in contrast to high-income countries, with an incidence ranging from 0.56 to 2 per 100 000.^7^

The clinical symptoms and signs of intracranial masses should never be underestimated, no matter how subtle, as they are clues that assist the clinicians in performing appropriate radiological investigations.

MRI is the investigation of choice in children with intracranial masses. The advantages of MRI include a lack of ionising radiation (when compared to computed tomography [CT] scans), and the best contrast resolution, especially with higher magnetic field scanners such as 3.0 Tesla (3.0T) scanners.^8^ Advanced imaging techniques, such as dynamic intravenous post-contrast evaluation, diffusion-weighted imaging (DWI), functional imaging, susceptibility-weighted imaging (SWI) and magnetic resonance spectroscopy (MRS), significantly improve diagnostic sensitivity and specificity.^9^

To our knowledge, no previous studies have reported on the MRI characteristics of intracranial masses in the paediatric population in KwaZulu-Natal (KZN), South Africa. The purpose of this study is to report these characteristics in paediatric patients managed at a tertiary state referral hospital.

## Research method and design

The study was performed at Inkosi Albert Luthuli Central Hospital, which is a tertiary hospital located in Durban, KZN. Inkosi Albert Luthuli Central Hospital is one of three hospitals that perform MRIs in the province of KZN that houses both the departments of neurosurgery and paediatric neurology, in a province of approximately 11 million.^10^ This was a retrospective, analytic, cross-sectional study design conducted between 01 January 2010 and 31 December 2016.

The clinical and radiological data of paediatric patients referred to the Radiology Department for an MRI scan who fulfilled our inclusion criteria were obtained from the hospital information system (Meditec). Radiology reports and image archives were obtained from the radiology information system and picture archiving and communication system. We included all children from birth to 18 years old with reported intracranial masses. Patients above the age of 18 years, children with MRI scans not performed at our institution, patients with no formal radiology reports and patients with intracranial masses secondary to trauma, congenital and vascular masses were excluded from the study.

The data collected included demographics, clinical features and MRI characteristics. All the studies were acquired on 3.0 Tesla Skyra Siemens MRI scanner. The scanning protocol for conventional sequences included T1-weighted (T1W), T2W, fluid-attenuated inversion recovery (FLAIR) and T1W post-contrast images. Susceptibility-weighted imaging was performed where haemorrhage and calcifications were suspected, and DWI was performed whenever it was deemed necessary by the consultant radiologist. Magnetic resonance spectroscopy was not routinely performed.

The data frequencies relating to the demographics and spectrum of masses were analysed. The analysis of the MRI characteristics was documented and correlated with the histological findings. Further correlation with the mass topography and clinical presentation was made. All analyses were performed using SPSS version 25 software.

### Ethical considerations

Ethics approval for this study was granted by the Biomedical Research Ethics Committee (BREC) of the University of KwaZulu-Natal (Ref. No. BE027/18).

## Results

**FIGURE 1:**
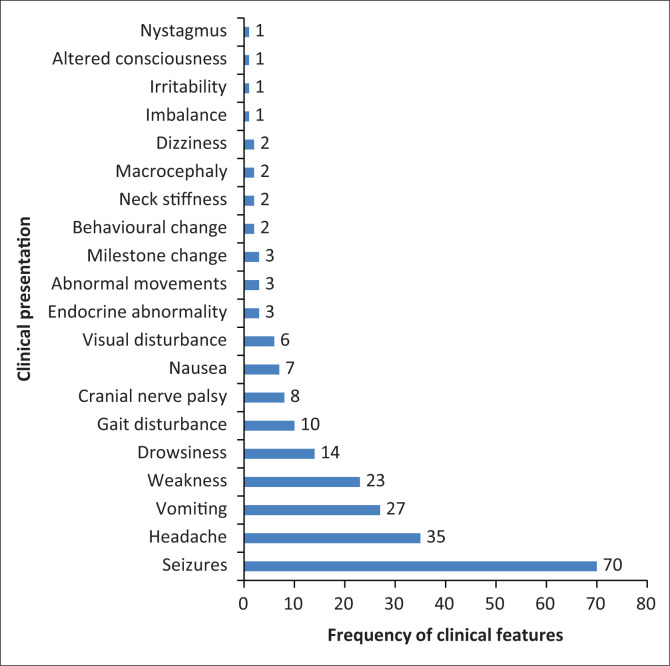
The clinical presentation of paediatric patients with intracranial masses.

A total of 931 MRI brain scans were performed during the 6-year period. One hundred and seven scans fulfilled the inclusion criteria. There were a total of 92 (86%) primary brain tumours and 15 (14%) infective or inflammatory masses. The majority of the children were females (*n* = 61, 56%) with a mean age of 12 ± 4.52 (range, 3–18 years). The most common presenting symptom was seizures (65.4%). Of the 70 patients who presented with seizures, 30 (43%) had masses located in the supratentorial compartment and 40 (57%) in the infratentorial compartment. The clinical presentations are demonstrated in Figure 1.

**FIGURE 2:**
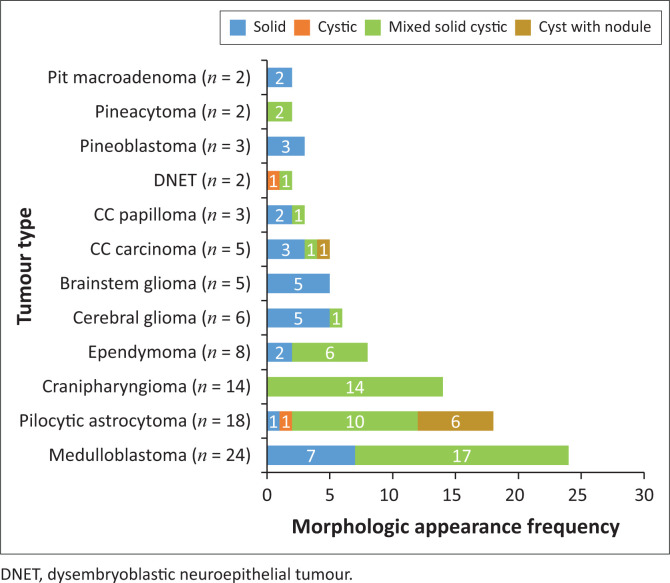
Morphologic appearance of paediatric brain tumours.

**FIGURE 3:**
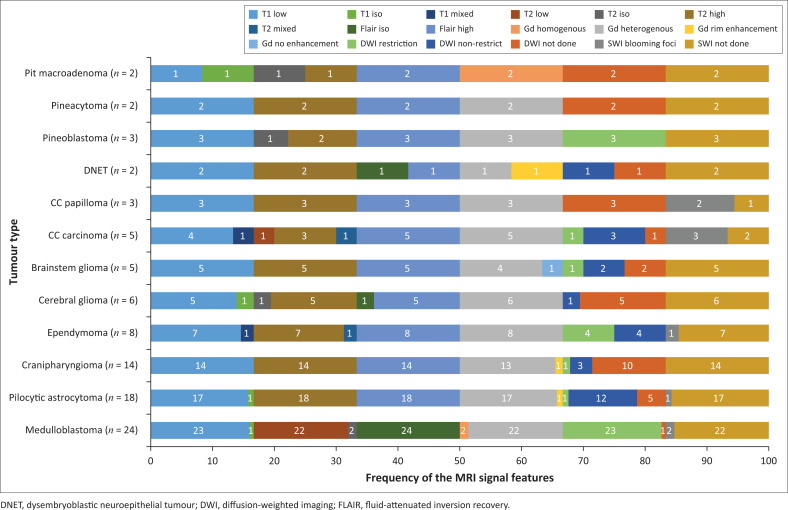
Magnetic resonance imaging features of paediatric brain tumours.

**FIGURE 4:**
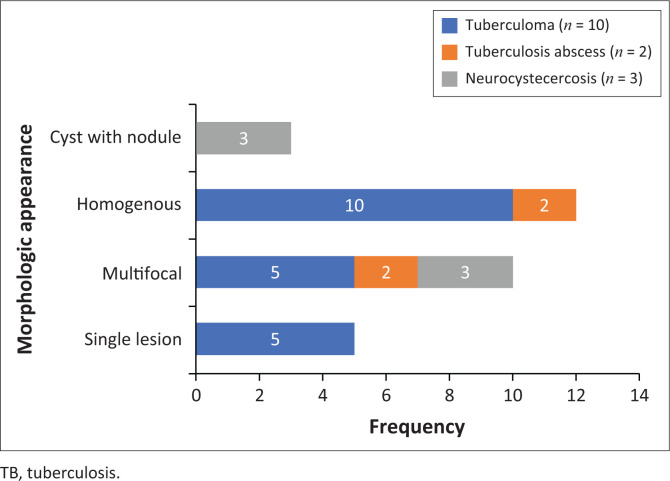
Morphologic features of paediatric infective or inflammatory intracranial masses.

**FIGURE 5:**
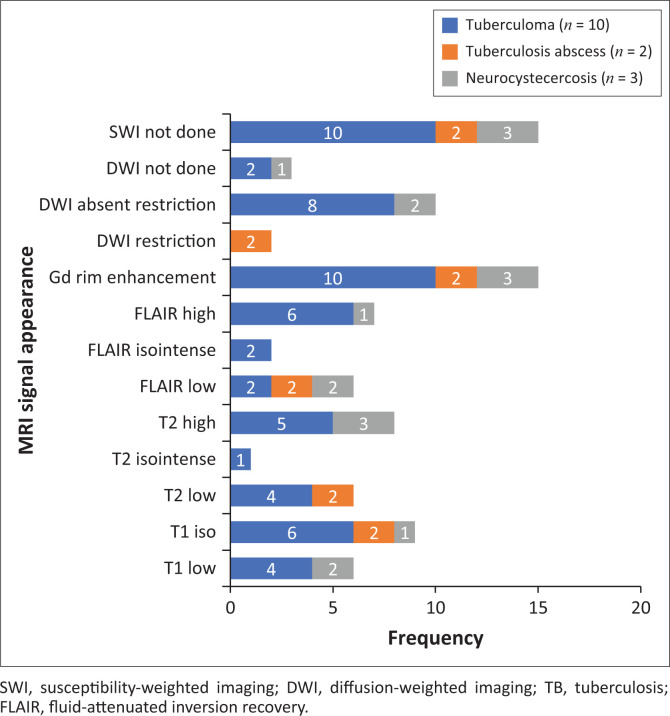
Magnetic resonance imaging features of paediatric infective or inflammatory intracranial masses.

The locations and diagnoses of the intracranial masses are summarised in Table 1. Magnetic resonance imaging features of the intracranial masses are shown in Figures 2, 3, 4 and 5. The associated features on MRI are also displayed in Table 2.

**TABLE 1:** Intracranial mass diagnosis, location and age groups.

Type of mass	Total (*N* = 107)		Age 0–1 years	Age 2–4 years	Age 5–10 years	Age 11–18 years
	*N*	%				
**Supratentorial Tumours**†						
Craniopharyngioma	14	13.1	0	1	1	12
Low-grade glioma	6	5.6	0	0	2	4
Ependymoma	4	3.7	0	0	0	4
Pilocytic astrocytoma	4	3.7	0	0	1	3
DNET	2	1.9	0	0	0	2
Choroid plexus papilloma	3	2.8	0	0	2	1
Choroid plexus carcinoma	5	4.7	0	1	2	2
Pineoblastoma	3	2.8	0	0	1	2
Pineocytoma	2	1.9	0	0	0	2
Pituitary macroadenoma	2	1.9	0	0	0	2
Total	45	42.1	0	2	9	34
**Supratentorial Infective or inflammatory masses**‡						
Tuberculous abscess	2	1.9	0	2	0	0
Tuberculoma	6	5.6	0	0	0	6
Neurocysticercosis	3	2.8	0	0	1	2
Total	11	10.3	0	2	1	8
**Infratentorial Tumours**§						
Medulloblastoma	24	22.4	0	2	6	16
Pilocytic astrocytoma	14	13.1	0	0	3	11
Brainstem glioma	5	4.7	0	0	2	3
Ependymoma	4	3.7	0	2	1	1
Total	47	43.9	0	4	12	31
**Infratentorial Infective or inflammatory masses**¶						
Tuberculoma	4	3.7	0	0	1	3
Total	4	3.7	0	0	1	3

DNET, dysembryoplastic neuroepithelial tumour.

† , No histological reports in four cases of low-grade glioma and one case of pineocytoma.

‡ , Number of infective or inflammatory masses histologically confirmed: only two cases of tuberculoma.

§ , All infratentorial tumours had histological reports except three cases of brainstem glioma.

¶ , Number of infective or inflammatory masses histologically confirmed: only one case of tuberculoma.

**TABLE 2:** Intracranial masses with pathological features.

Type of mass	Supratentorial masses		Infratentorial masses	
	*N*	%	*N*	%
**Total count**				
Tumour	45	42.1	47	43.9
Infection	11	10.3	4	3.7
**Vasogenic oedema**				
Tumour	31	68.9	46	97.9
Infection	11	100.0	4	100.0
**Hydrocephalus**				
Tumour	35	77.8	45	95.7
Infection	1	9.1	2	50.0
**Herniation**				
***Subfalcine herniation***				
Tumour	15	33.3	-	-
Infection	3	27.3	-	-
***Tonsillar herniation***				
Tumour	1	2.2	21	44.7
Infection	-	-	1	25.0
***Uncal herniation***				
Tumour	1	2.2	-	-

Paediatric central nervous system masses.

## Discussion

According to numerous case series studies, medulloblastoma is the commonest infratentorial tumour, which is a similar finding in this study.^5,6^ A South African study conducted in Johannesburg also reported similar results.^3^ Tuberculomas, in the current study, commonly occurred in the supratentorial compartment, which differed from the literature where the infratentorial compartment was most common.^11,12^

### Clinical presentation

We noted a variable pattern of clinical presentations, but the most frequent presentations, regardless of the mass characteristics or mass location, were seizures (65.4%) and headaches (33%).

The majority of our patients who presented with seizures had tumours located in the infratentorial compartment, which is contrary to the reported literature, which states that seizures are more common with supratentorial tumours than infratentorial tumours.^13^ However, almost all of our infratentorial tumours had associated features of raised intracranial pressure (ICP), which is known to have a non-specific association with seizures.

The majority (80%) of inflammatory masses also presented with seizures, which is a known clinical presentation.^14,15^ Tumour-related seizures result from metabolic, neurotransmitter and morphologic changes in the peritumoural brain, as well as the presence of peritumoural products, gliosis and necrosis. Neurological excitation from pro-inflammatory signals causes seizures from inflammatory masses.^16^

### Tumour characteristics and location

#### Infratentorial tumours

These were common in the 11–18 year-old age group, contrary to the other studies, that report an age group of less than 3 years.^17^

The most common infratentorial tumours were medulloblastoma (24/47, 51.1%) and pilocytic astrocytoma (14/47, 30.0%), which are consistent with the literature.^3,5,6^

**FIGURE 6:**
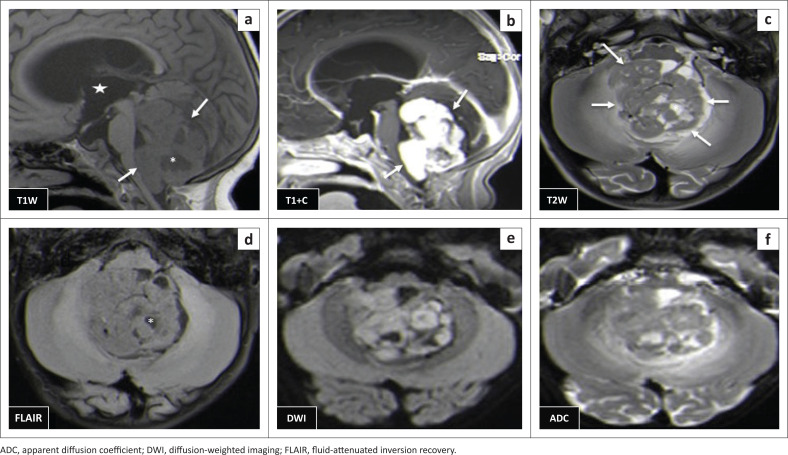
(a–f) Medulloblastoma, predominantly centred in the fourth ventricle. The mass is predominantly solid (arrows) with tiny cystic areas (*) and avid enhancement of the solid areas (b). Two images (c, d) show T2 and FLAIR low signal of dense hypercellular solid areas and fluid restriction on DWI and ADC map. Supratentorial hydrocephalus is also seen (star).

This study found medulloblastoma to have the typical described morphology of heterogeneity with mixed solid and cystic areas, the solid areas appearing hypointense to grey matter on T2WI, restriction on DWI owing to the dense cellularity, heterogeneous enhancement on post-IV gadolinium T1WI and occasional blooming artefact from calcification on SWI.^7^ These features matched the majority of findings as depicted in Figures 3 and 6, except that SWI was only performed in two cases, where there was evidence of calcification and blooming artefact.

**FIGURE 7:**
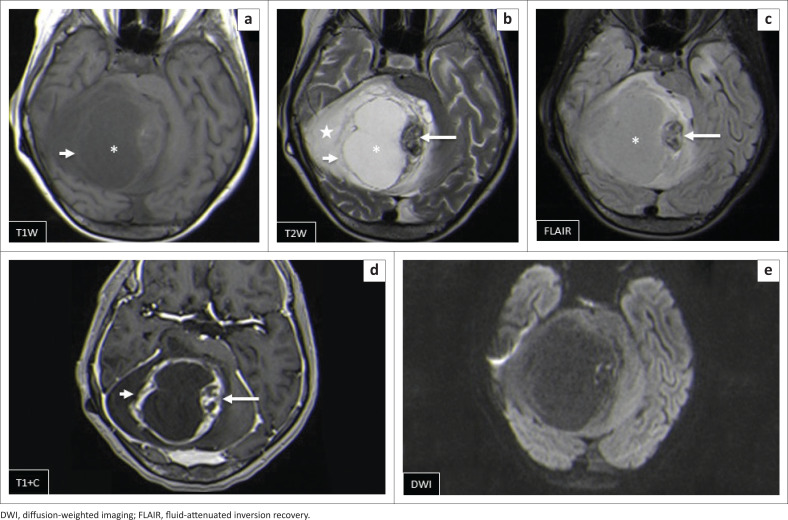
(a–e) Right cerebellar pilocytic astrocytoma. This is a large cystic mass (*) with an eccentric solid nodule (long arrow). There is enhancement of the tumour wall (short and long arrows [d]) and surrounding vasogenic oedema (star). There is no remarkable diffusion restriction (e).

Pilocytic astrocytoma was the second common intracranial tumour in the infratentorial compartment. The majority appeared heterogeneous with solid and cystic components that demonstrated heterogeneous post-contrast enhancement. According to the literature, this is not typical of the classic appearance of a rim-enhancing cyst with an enhancing mural nodule. Other appearances include a non-enhancing cyst wall with an enhancing mural nodule, a solid mass with necrotic centre (16%) and a solid mass with minimal or no cystic component (17%).^6,18,19^ These three appearances were not evident in this series. Appearances on additional sequences did not differ from the already-described literature. The cystic component was isointense to cerebral spinal fluid (CSF) on T1W and T2W sequences, and hyperintense on FLAIR. The solid component was hypo- to isointense on T1WI, hyperintense on T2WI and FLAIR and post-contrast showed variable enhancement patterns^19^ (Figure 7). At DWI, pilocytic astrocytomas do not restrict because of the low tumour cellularity.

**FIGURE 8:**
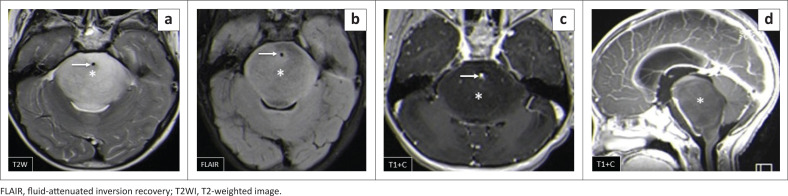
(a–d) Typical diffuse intrinsic pontine glioma that expands the pons (*) and encases the patent basilar artery (arrow). It is hypointense on T1WI, hyperintense on T2WI and FLAIR and does not enhance post-gadolinium. There was no restricted diffusion (not shown).

Brainstem gliomas and the ependymomas were the least common infratentorial tumours. The findings of brainstem gliomas in this study matched the described literature of being hypointense on T1WI and hyperintense on T2WI and FLAIR. Post-contrast imaging varied from no enhancement to minimal, patchy or heterogeneous (Figure 8) enhancement. Absent restricted diffusion is noted at DWI.^6^

**FIGURE 9:**
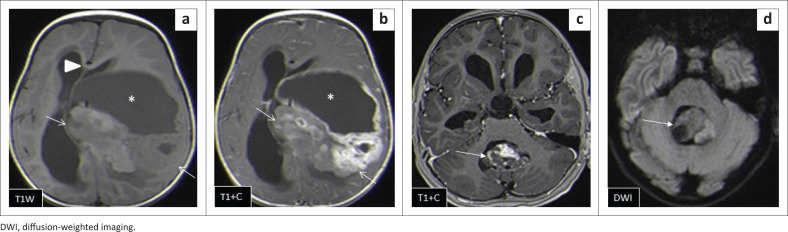
(a, b) Heterogeneously enhancing supratentorial ependymoma with solid (arrows) and cystic (*) components and heterogeneously enhancing solid areas. Associated subfalcine herniation with midline shift is seen (arrowhead). Two images in another patient (c, d) show an infratentorial ependymoma with a similar heterogeneous imaging appearance, which is lacking restricted diffusion in (d).

Ependymomas can occur anywhere in the central nervous system (CNS). They arise along the ventricular walls in the brain. They are reported to be most common in the fourth ventricle, typically heterogeneous, mixed, solid and cystic tumours, with heterogeneous enhancement and blooming artifact secondary to calcifications.^6^ In our small cohort, we observed imaging appearances matching the descriptions in the literature (Figures 9 a–d).

#### Differentiating infratentorial tumours

Common posterior fossa mass differentials in paediatric patients include medulloblastoma, ependymoma and pilocytic astrocytoma. Medulloblastoma and ependymoma share several morphologic features of a solid and cystic nature and some differences in MRI signal appearances. Like most tumours with low cellularity, the ependymomas are commonly of T1 low signal and T2 and FLAIR high signal with variable DWI signal in contrast to earlier described medulloblastoma features. Location and local tumour growth behaviour offer additional input in making the distinction. Medulloblastoma grows into the fourth ventricle from the vermis whilst ependymoma grows within the fourth ventricle, hence expanding the ventricle. The ependymoma typically extends into the foramina or Luschka and Magendie, which is not usual for medulloblastoma. Pilocytic astrocytoma, by contrast, is typically located in the cerebellar hemisphere and is commonly cystic with a mural nodule.^20^

#### Supratentorial tumours

**FIGURE 10:**
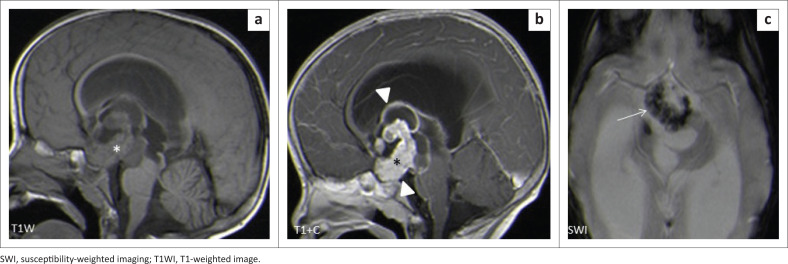
(a–c) Heterogeneous craniopharyngioma in the sellar and suprasellar region with solid and cystic components (triangles). The solid components enhance post-gadolinium (*), and SWI shows blooming artefact (arrow) secondary to calcifications.

The most common tumour in the supratentorial compartment was craniopharyngioma. It is commonly a suprasellar mass but can arise anywhere between the third ventricle and the pituitary gland. The adamantinomatous craniopharyngioma generally appears as a mixed solid and cystic mass with calcifications. The papillary type is commonly solid with fewer calcifications.^21,22^ Solid components may appear iso- to hypointense on T1WI, iso- to hyperintense on T2WI and hyperintense on FLAIR imaging in relation to grey matter. The cystic component can be hypointense or hyperintense on T1WI, owing to the presence of cholesterol, proteinaceous fluid or methaemoglobin. On FLAIR imaging, it is hyperintense. Calcifications on the solid components or the rim of the cyst appear hypointense on T2WI and demonstrate blooming artefacts on SWI.^6^ Post-contrast, the solid component enhances, and the cystic component demonstrates rim enhancement. On DWI, they do not restrict.^21^ The findings in our series matched those typically described appearance (Figures 3 and 10).

Differentials for craniopharyngioma include a pituitary adenoma, hypothalamic or optic pathway glioma and a Rathke’s cleft cyst. A pituitary adenoma is usually an intrasellar mass that expands the sellar with suprasellar extension, has no calcifications and possesses variable cystic components. Hypothalamic or optic pathway gliomas may have cystic areas but are largely dominated by solid components and rarely have an intrasellar component or calcifications. Large Rathke’s cleft cysts are usually easier to differentiate from the smaller ones because they usually do not have a solid component or calcifications and do not enhance post-contrast.^21^

**FIGURE 11:**
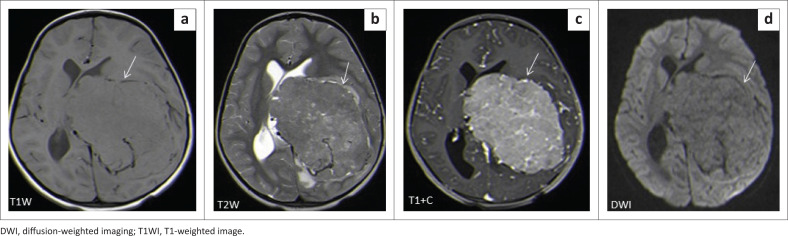
Choroid plexus carcinoma (a–d). There is a large T1 and T2 isointense mass with intense heterogeneous enhancement within the left lateral ventricle causing midline shift to the right. It shows no restriction at DWI.

Choroid plexus carcinoma (CPC) and choroid plexus papilloma (CPP) are commonly located in the atrium of lateral ventricle (50%), followed by the fourth and third ventricles, with an incidence of 40% and 5%, respectively.^23^ In this study, both tumours were located in the lateral ventricles, followed by the third ventricle (Figure 11) and the extra-ventricular location of CPC and CPP was found in the frontal lobe (CPC) and the parieto-occipital lobe (CPP). The extra-ventricular location of these tumours is rare. Both tumours were classically heterogeneous with solid and cystic components as well as calcifications. They typically enhance avidly on post-contrast imaging because of their high vascular nature. The signal intensities of both tumours are iso- to hypointense on T1WI and iso- to hyperintense on T2WI.^23^ Most of our findings match the above signal characteristics on pre- and post-gadolinium sequences. On SWI, most had calcification related blooming artefacts. The restricted diffusion feature can be used to differentiate CPC from CPP; however, in our cohort, DWI was only performed in CPC cases, and most demonstrated no restriction, except for one case.

The differential diagnosis for intraventricular choroid plexus tumours includes ependymoma, central neurocytoma and subependymal giant cell astrocytoma. Central neurocytoma is usually located in the body of the lateral ventricle arising from the septum pellucidum or ventricular wall. It frequently has multicystic components and may have calcifications as well as areas of haemorrhage. It is hypointense on T1WI, hyperintense on T2WI and has a variable enhancement pattern. It is common in elderly patients.^23^ A subependymal giant cell astrocytoma occurs almost exclusively in association with tuberous sclerosis located in the foramen of Monro, appearing as a lobulated enhancing mass with a T1 low signal and T2 high signal and containing calcifications.

#### World Health Organization classification of central nervous system tumours

The World Health Organization (WHO) classification of CNS tumours is a worldwide well-recognised system of classifying CNS tumours that is based on histological and molecular tumour characteristics. It is worth noting that DWI offers a potential volatile tool as a practical imaging guide to the WHO tumour grade. Mustafa et al.^24^ and several other authors noted significant differences of paediatric CNS tumour water diffusion properties utilising apparent diffusion coefficient (ADC) map values, which somewhat correlate with the WHO tumour grade. Significant differences regarding fluid diffusion restriction were noted in pilocytic astrocytoma, ependymoma, medulloblastoma and brainstem glioma. Pilocytic astrocytoma, a known WHO Grade 1 tumour, showed no fluid restriction (high ADC map values) in contrast to a medulloblastoma, a known WHO Grade 4 tumour that had restricted diffusion (very low ADC map values).

Tumours with low cellularity (i.e. no diffusion restriction) and less aggressiveness, including pilocytic astrocytoma, CPP, pineocytoma and craniopharyngioma, are known to be classified as WHO Grade 1 tumours. The moderately cellular ependymoma, a known WHO Grade 2 tumour, has variable restricted diffusion according to imaging literature.^24,26^ It was only between pilocytic astrocytoma and ependymoma where Mustafa et al.^24^ did not find a significant difference on ADC map values. Further contrasts of other densely cellular tumours with restricted diffusion such as CPC and pineoblastoma are known grades 3 and 4 WHO classifications, respectively.

Several studies in the literature have demonstrated high ADC map values (no restriction) in WHO grade 1 and 2 gliomas and low ADC map values (fluid restriction) in WHO grade 3 and 4 gliomas.^24,25,26^

### Infective or inflammatory masses

**FIGURE 12:**
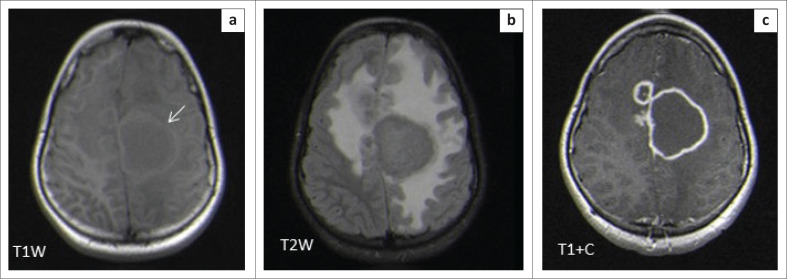
Caseating tuberculoma with a solid centre (a–c). Two high parietal masses that are isointense to grey matter on T2WI with a slightly hyperintense rim (arrow), hypointense on T1WI and demonstrate rim enhancement post-gadolinium. Note the significant perilesional oedema with increased white matter signal intensity (b).

The most common infective masses were secondary to tuberculosis (TB). Tuberculosis and malaria are reported as common infections in sub-Saharan Africa.^16^ These parenchymal TB masses manifest as tuberculomas and TB abscesses. Tuberculomas are the most common manifestation of CNS TB, and, in paediatric patients, they are said to be more common infratentorially within the cerebellum.^27^ In contrast, most of our patients had granulomas in the supratentorial region within the cerebral lobes. Tuberculomas have different stages, which make them appear differently on MRI sequences. These stages include a non-caseating granuloma, a caseating granuloma with a solid centre, a caseating granuloma with central liquefaction and a calcified granuloma. A non-caseating granuloma is iso- to hypointense on T1WI, hyperintense on T2WI, hyperintense on FLAIR and has solid enhancement on post-contrast imaging. A caseating granuloma is iso- to hypointense with a hyperintense rim on T1WI, hypointense on T2WI, hyperintense on FLAIR and shows rim enhancement on post-contrast imaging (Figures 4 and 12). A caseating granuloma with central liquefaction is iso- to hypointense with a hyperintense rim on T1WI, a hypointense rim with a central hyperintense signal on T2WI, with partial suppression on FLAIR and rim enhancement on post-contrast images. All these active granulomas show no restriction on DWI, except for the caseating granuloma with central liquefaction, which may restrict.^27^

Tuberculous abscesses were not common in our series. They are mostly seen in immunocompromised patients. The appearance in the current series was that of hypo-intensity on T1WI, heterogeneous hyperintensity on T2WI, variable suppression on FLAIR and rim enhancement on post-contrast imaging. At DWI, they demonstrate restricted diffusion. It is vital to differentiate granulomas from abscesses, because their management is different. The management of granulomas is medical, with anti-tuberculosis drugs, whilst abscesses often require both surgical drainage and medical therapy.^27^

The differential diagnosis of tuberculosis includes a variety of rim-enhancing masses, which may be another infection, such as toxoplasmosis and neurocysticercosis, as well as malignancy, such as CNS lymphoma, a primary tumour (glioblastoma multiforme) or metastasis.^27^ The associated features, such as meningeal enhancement, basal ganglia infarcts and communicating hydrocephalus, are more likely to occur with TB masses compared to the described differential. Toxoplasmosis can be a single or multifocal masses, with a predilection for basal ganglia and grey–white matter junctions. Toxoplasmosis masses are somewhat small with an irregular rim of enhancement and a high DWI signal. Neurocysticercosis can be located at the grey–white matter junctions, in the subarachnoid CSF spaces and within the ventricles. There are various stages affecting MRI features; however, the typical small cystic appearance with an internal eccentric scolex offers more confidence in making the diagnosis. Primary CNS lymphoma tends to be a larger infiltrating enhancing solid mass lining the ventricular wall, although it can have a necrotic centre. The high DWI signal within the mass is related to the dense cellularity in lymphomas.

The use of MR spectroscopy imaging can help discriminate tumours (lymphoma and metastasis) from infective masses as tumours demonstrate an elevated choline peak.^28,29^

### Pathological features of intracranial masses

#### Oedema

Vasogenic oedema was present with all the infective masses and most tumours (*n* = 77/92 = 83.7%). The most common tumours associated with the oedema were in the infratentorial compartment.

These results correlate with literature that reports that both brain pathologies, tumours and infective masses, cause damage to the blood-brain barrier (BBB). In tumours, BBB damage is secondary to tumour angiogenesis. Infections actually result in both vasogenic and cytotoxic oedema (combined oedema), with cytotoxic oedema initially occurring from the derangement of the adenosine-dependent triphosphate (ATP) –transmembrane sodium-potassium and calcium pumps. Initially, the BBB is spared, but with increasing insult severity, cell death occurs, which then damages the BBB and results in vasogenic oedema.^30^

#### Hydrocephalus

Within the infratentorial group, the majority of tumours, (*n* = 45/47, 96%) and infections, (*n* = 2/4, 50%) were associated with hydrocephalus, compared to the supratentorial group (tumours, *n* = 35/45, 77.8%; infections, *n* = 1/11, 9%). This is not surprising because of the anatomical relationship of infratentorial masses with CSF drainage pathways, which result in obstruction of CSF flow attributable to compression of the fourth ventricle. This finding corresponds with the literature.^31^ Sindi Lam et al. reported that hydrocephalus occurs in 71% – 90% of infratentorial tumours.^32^ Hydrocephalus in supratentorial masses is secondary to obstruction of the third ventricle and cerebral aqueduct.

#### Herniation

Thirty-nine percent of the patients had associated herniation, secondary to raised ICP. Most masses were located in the infratentorial compartment (*n* = 22/51, 43.1%), causing tonsillar herniation. This occurs when there is increased pressure in the posterior fossa, causing herniation of the cerebellar tonsil through the foramen magnum.^33^ This can be attributable to the size of the mass (large mass) and the presence of significant associated vasogenic oedema. Supratentorial masses were associated with subfalcine herniation (*n* = 18/56, 32.1%), where increased pressure caused herniation of the cingulate gyrus beneath the falx cerebri.^33^

## Limitations of the study

The limitation of this study was the retrospective design and the single centre study. However, all paediatric patients with intracranial mass masses were managed at a single tertiary centre in the province of KZN. A larger cohort may have provided more information.

## Conclusion

In this series, the supratentorial compartment was the most common site for intracranial masses overall. The most common tumour was, however, a medulloblastoma, located in the infratentorial compartment. This information is an important guide when developing the differential diagnosis of common intracranial masses in paediatric patients referred to our institution and in planning further management.
